# Establishment of discrete reference interval and next-generation reference interval for copper and zinc during pregnancy using real-world data

**DOI:** 10.1016/j.heliyon.2024.e33856

**Published:** 2024-06-29

**Authors:** Lihua Guan, Chaochao Ma, Liling Lin, Ling Qiu

**Affiliations:** a Department of Laboratory Medicine, Peking Union Medical College Hospital, Peking Union Medical College & Chinese Academy of Medical Science, Beijing 100730, China; b State Key Laboratory of Complex Severe and Rare Diseases, Peking Union Medical College Hospital, Peking Union Medical College & Chinese Academy of Medical Science, Beijing 100730, China; c Department of Occupational and Environmental Health Sciences, School of Public Health, Peking University, Beijing, 100191, China

**Keywords:** Pregnant women, copper, Zinc, Discrete reference interval, Next-generation reference interval

## Abstract

**Background:**

Copper and zinc are two trace elements that are essential to maintain normal pregnancy and fetal development. But only few research established specific reference intervals (RIs) for pregnant women. In this study, we aim to establish discrete RIs and next-generation RIs for copper and zinc during pregnancy by real-world data.

**Method:**

We retrospectively collected 710 healthy pregnant women and 300 age-matched non-pregnant women attending the hospital and compared copper and zinc levels among them. Further, we analyzed multiple factors (gestational age, maternal age, and parity) that may affect copper and zinc during pregnancy by multivariate regression. Two types of reference intervals (RIs) of copper and zinc for pregnant women were established: discrete reference intervals (RIs) by non-parametric method and next-generation RIs by Generalized Additive Models for Location, Scale, and Shape (GAMLSS) model.

**Result:**

Copper levels were higher (median: 1st trimester: 1203.00 μg/L, 2nd trimester: 1818.00 μg/L, 3rd trimester: 1795.00 μg/L) than in non-pregnant women (median: 900.00 μg/L, p＜0.001), whereas zinc levels were lower in pregnant women (median: 1st trimester: 836.00 μg/L, 2nd trimester: 639.00 μg/L, 3rd trimester: 618.00 μg/L) than in non-pregnant women (median: 767.00 μg/L, p＜0.001). Additionally, copper and zinc levels varied among trimesters. Moreover, copper and zinc were affected significantly by gestational age but maternal age only had a weak effect on them. Based on the effect of gestational age, we established discrete RIs partitioned by trimesters and next-generation RIs for copper and zinc respectively during pregnancy.

**Conclusion:**

Taken together, this research elucidated the remarkable effect of gestational age on copper and zinc during pregnancy. The next-generation RIs visualized trends and subtle changes in copper and zinc levels during pregnancy. The discrepancy between discrete RIs and next-generation RIs suggested a more detailed continuous RIs could be considered for pregnant women.

## 1 Introduction

Copper and zinc, two trace elements, are mainly obtained from diets and interact with each other. They are involved in the composition of many enzymes, oxidative stress, etc, which are crucial for human health, especially pregnant women. In pregnant women, copper and zinc deficiencies or excesses may lead to miscarriage, preterm labor, or poor fetal development [1,2]. Herein, accurate monitoring of copper and zinc levels is vital to maintain normal pregnancy and development of the fetus. During pregnancy, women may experience dramatic physiological and metabolic changes, such as maternal-fetal transport and hormone effects, as well as dietary changes, copper and zinc levels may be different from non-pregnant women [3], showing the necessity to establish specific reference intervals (RI) for pregnant women. More importantly, with the advancement of mass spectrometry, inductively coupled plasma mass spectrometry (ICP-MS), a more sensitive and specific method, has been a substitute method for the traditional atomic absorption photometric method (AAS) for the detection of trace elements. Different results caused by variations in assay suggest the requirement to establish method-specific RIs [4]. However, only few articles established specific RIs for copper and zinc in pregnant women, particularly based on ICP-MS [5,6]. As such, it is important to establish specific RIs for copper and zinc based on ICP-MS during pregnancy.

Traditionally, researchers utilized discrete RIs for analytes in pregnant women that are partitioned by trimesters and other influence factors. However, discrete RIs may simplify the complex relationship between analytes and influence factors, and the set of thresholds affects greatly on different RIs among studies. For example, variations of trimester-specific RIs in different studies may partly due to differences of the definition of trimesters [5,6]. More importantly, changes within the group in discrete RIs are overlooked and may result in wrong clinical decisions. Conversely, next-generation RIs, or continuous RIs, enable a more detailed and more sensitive depiction of changes in analytes than discrete RIs, particularly age-dependent changes. Additionally, next-generation RIs have been widely used in recent years and are highly advantageous for various analytes [[7], [8], [9], [10], [11]] [[7], [8], [9], [10], [11]]. Among algorithms for estimating next-generation RIs, Generalized Additive Models for Location, Scale, and Shape (GAMLSS) employs parameters of the distribution (location, scale, skewness and kurtosis) to develop models and then estimate next-generation RIs [12]. Currently, the GAMLSS model has been widely used in establishing next-generation RIs and presents with superior applicability in clinical practice [[13], [14], [15]] [[13], [14], [15]].

In this study, we used real-world data to compare the differences in copper and zinc between pregnant women and non-pregnant women. Further, we investigated the impact of multiple factors on copper and zinc during pregnancy. Finally, we established discrete trimester-specific RIs for copper and zinc by the non-parametric method and as well as continuous gestational-age based RIs by the GAMLSS model. It is expected that this study will plot the trend of copper and zinc during pregnancy, which may help physicians monitor copper and zinc status, and provide dietary modifications and therapeutic recommendations for the prevention and reversal of copper and zinc abnormalities. Moreover, dynamic trends of copper and zinc can help better understand mechanisms on changes in copper and zinc during pregnancy.

## 2 Method and materials

### 2.1 Data source

We retrospectively analyzed data of pregnant women who were tested copper and zinc at the Peking Union Medical College Hospital from April 2022 to August 2023. Participants were enrolled in the reference dataset if they met the following inclusion and exclusion criteria:

Inclusion criteria.

(1) Individuals had serum copper and zinc results and demographic information (age, last menstrual period (LMP), etc.);
(2) Age≥18 years;

Exclusion criteria.

**Fig. 1:**
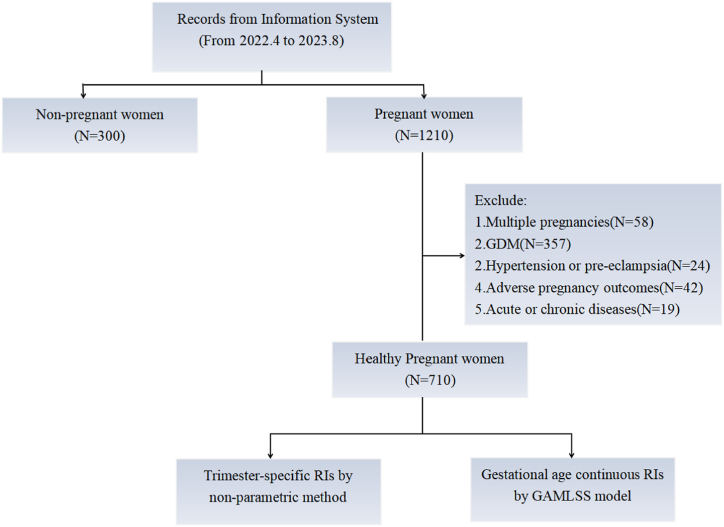
Flow chart of the study.

(1) Adverse pregnancy outcomes including abortion, fetal malformation, still birth, and embryo arrest;
(2) Pregnancy complications including gestation diabetes, preeclampsia;
(3) History of acute or chronic diseases, including circulatory diseases, respiratory diseases. digestive diseases, urinary diseases, autoimmune diseases, hematological diseases, acute and chronic infections;

Finally, a total of 710 healthy pregnant women were included in this study. Trimesters were defined as 1st trimester (<14w), 2nd trimester (14-＜28w), 3rd trimester (≥28w) (Gestational age was calculated by ultrasound data and/or the first day of LMP). Meanwhile, we enrolled 300 non-pregnant women over the research period (Fig. 1).

### Ethical approval

This retrospective study was approved by the Ethics Committee of the Peking Union Medical College Hospital of the Chinese Academy of Medical Sciences (K4670). Owing to the retrospective nature of this study, informed consent was not required.

### 2.2 Analytical performance of analytes and quality control

Results of plasma copper and zinc levels were measured using a YS EXT 8600MD inductively coupled plasma mass spectrometry (ICP-MS) (Yingsheng, China), with corresponding standards, internal standards, and quality controls supplied by the manufacturer. Fasting blood samples were collected through venipuncture and blue-caped sodium citrate anticoagulation tubes (Vacuette, Greiner Bio-One GmbH, Frickenhausen, Germany). Following venipuncture, samples were centrifuged within 2 h of collection to obtain plasma. Once plasma was collected, the samples were tested immediately.

Quality assurance in this study was composed of two aspects: data generation and data analysis. During the data generation phase, the parameter settings, calibration, and detection procedures were conducted according to the standard operating procedures in the instructions book. Three levels of quality controls were conducted before sample testing once a day and samples were detected only after quality control was qualified, making sure the results obtained over the research period were accurate and reliable. Over the time course of the study, the mean total imprecision (coefficient of variation) of copper was 2.492 % in the low level (a mean value of 723.59 μg/L), 2.210 % in the media level (a mean value of 1429.2 μg/L), and 4.176 % in the high level (a mean value of 2431.32 μg/L). For zinc, the mean total imprecision was 5.337 % in the low level (a mean value of 461.29 μg/L), 4.648 % in the media level (a mean value of 706.29 μg/L), and 4.219 % in the high level (a mean value of 1387.6 μg/L) (The allowable coefficient of variation is 15 %). Furthermore, Our laboratory receives yearly inspections by the National Center for Clinical Laboratories and the College of American Pathologists every 2 years, certifying the reliability and precision of our data. As for data analysis and programming, each code was reviewed, checked, and tested by two separate individuals at the same time. The dual-review process assured the results were analyzed robustly and consistently.

### 2.3 Data cleaning and statistical analysis

All data were stored in Microsoft Excel 2016 and were analyzed with IBM SPSS Statistics (Version 26.0), MedCalc software (version 20.022), and R language (version 4.3.2). The Kolmogorov-Smirnov test was used to evaluate the normality of the data distribution. Continuous variables with a normal or approximately normal distribution were expressed as mean (standard deviations) and the outliers were identified by the Tukey method [16,17]. Before outliers were excluded, Box-cox transformation was used to improve the normality of data. The Box-cox transformation was conducted through the formula below (The λ and c values were deduced by the maximum likelihood algorithm) in the forecast package (version 8.21.1) of R software:

$$y=\left\{ \begin{array}{c} \left(x^{\lambda }-1\right)/\lambda ,\lambda \neq 0\\ \ln\left(x+c\right),\lambda =0 \end{array}\right.$$

The Tukey method recognized outliers by computing the 25th (Q1), 75th (Q3) percentiles, and interquartile range (IQR = Q3 − Q1). Outliers were below Q1 − 1.5 × IQR or above Q3 + 1.5 × IQR. The skewed distribution variables were presented as median (interquartile, IQR), and categorical variables are expressed as the number with a percentage. The Kruskall-Wallis test was used to compare the differences in demographic characteristics, and plasma copper and zinc levels across subgroups. The effects of gestational age, maternal age, and parity on copper and zinc were examined by multivariate regression analysis with standardization regression coefficient (β). A two-sided p-value＜0.05 was considered as statistically significant.

We utilized two types of methods to establish RIs for copper and zinc: discrete RIs by the non-parametric method, and continuous RIs by the GAMLSS method. As for the establishment of discrete RI, the Box-cox transformation was performed again when establishing RIs. Then we utilized the non-parametric method with MedCalc software [18,19]. RIs were calculated as the 2.5–97.5th percentile with a 90 % confidence interval (CI) for upper limits (ULs) and lower limits (LLs) [18]. Further, the Hoffmann method [[20], [21], [22]] [[20], [21], [22]], an indirect method capable of identifying healthy populations from mixed populations, was utilized to further establish discrete RI to demonstrate the enrolled healthy pregnant populations and the applicability of RIs established by non-parametric in this study to pregnant women (Supplement Table 2). Second, gestational-age based continuous RIs were established by the GAMLSS model. The Box-cox t distribution and cubic spline smoothing method with k = 3 (k is the number of effective degrees of freedom used in the model) was employed in the curve fitting procedure [12,23]. In each gestational age group, 2.5th, 25th, 50th, 75th,97.5th centiles were estimated through 4 distribution parameters (median(μ), variability(σ), kurtosis(τ), and skewness(υ)). Residual plots were used to evaluate whether simulation of kurtosis was required. RIs were also calculated as 2.5–97.5th percentile.

## 3 Result

### 3.1 Baseline information of enrolled participants

A total of 710 records from pregnant women and 300 records from non-pregnant women were included in this study. Pregnant women were further divided into three subgroups based on trimesters (1st trimester = 280, 2nd trimester = 248, 3rd trimester = 182). The median age was 33 among the four subgroups (P＞0.05). Maternal BMI varied among trimesters (Table 1).

**Table 1:** Basic information of pregnant women and non-pregnant women.

		Pregnant women				Non-pregnant women
		1st trimester	2nd trimester	3rd trimester	P value	
Number		280	248	182	\	300
Age (Year)		33 (30,36)	33 (31,35)	33 (31,35)	＞0.05	33 (29,38)
Gestation Age (Week)		7 (6,9)	21 (18,25)	32 (30,34)	＜0.05	\
BMI		21.80 (20.04,24.28)	23.31 (20.82,25.78)	24.20 (22.25,26.16)	＜0.05	
Parity	0	227 (81.1 %)	212 (85.5 %)	160 (87.9 %)	\	\
	1	48 (17.1 %)	31 (12.5 %)	20 (11.0 %)	\	\
	≥2	5 (1.8 %)	5 (2.0 %)	2 (1.1 %)	\	\

Data presented as Median(IQR).

### 3.2 Comparison of copper and zinc levels between pregnant and non-pregnant women

**Fig. 2:**
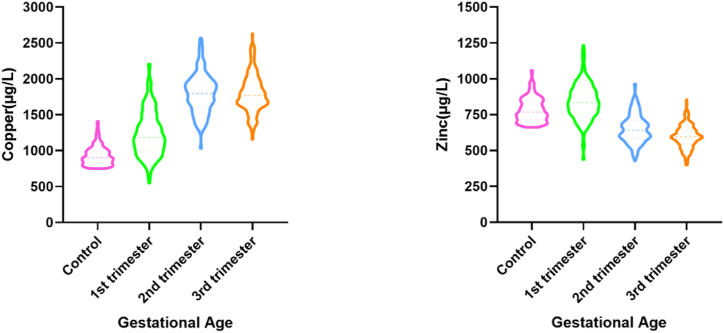
Comparison of copper and zinc among different subgroups. The violin plots show copper (left) and zinc (right) levels among different subgroups. Different color represent different subgroups (pink: control group; green:1st trimester group; blue: 2nd trimester group; orange:3rd trimester group). Violin plots show the density (width), median (center dot line), third quartile (top dot line), and first quartile (bottom dot line).

The profiles of serum copper and zinc in pregnant women and non-pregnant women subgroups are showed in Table 2 and Fig. 2. The median levels of copper during the 1st, 2nd, 3rd trimester were 1203.00 μg/L, 1818.00 μg/L, and 1795.00 μg/L, respectively, all of which were higher than copper levels in the non-pregnant women (900.00 μg/L). In pregnant women subgroups, copper levels were significantly higher in the 2nd trimester than 1st trimester (P＜0.001), while there is no significant differences between 2nd trimester and 3rd trimester (P＞0.05). For zinc levels, zinc levels differed across trimesters, with the median zinc levels were 836.00 μg/L, 639.00 μg/L, and 618.00 μg/L in the 1st, 2nd, and 3rd trimester, respectively, and were significantly lower than non-pregnant women (767.00 μg/L).

**Table 2:** Profiles of copper and zinc levels in different subgroups.

	Pregnant women			Non-pregnant women	P value
	1st trimester	2nd trimester	3rd trimester		
Copper (μg/L)	1203.00^a^^,^^d^^,^^e^ (1019.50–1465.50)	1818.00^b^^,^^d^ (1650.50–1978.00)	1795.00^c^^,^^e^ (1618.00–2002.00)	900.00^a^^,^^b^^,^^c^ (825.50,1001.75)	＜0.001
Zinc (μg/L)	836.00^a^^,^^d^^,^^e^ (762.25–917.00)	639.00^b^^,^^f^ (584.50–716.00)	618.00^c^^,^^e^^,^^f^ (567.00–694.00)	767.00^a^^,^^b^^,^^c^ (716.00,845.00)	＜0.001

Data presented as Median(IQR).

a . 1st trimester vs. Control, p＜0.05.

b . 2nd trimester vs. Control, p＜0.05.

c . 3rd trimester vs. Control, p＜0.05.

d 1st trimester vs. 2nd trimester, p＜0.05.

e . 1st trimester vs. 3rd trimester, p＜0.05.

f 2nd trimester vs. 3rd trimester, p＜0.05.

### 3.3 Impact on copper and zinc levels during pregnancy

To investigate possible influence factors on copper and zinc levels during pregnancy, we utilized multivariate regression with standard regression coefficient (β) to analyze the impact of gestational age, maternal age, and parity and results are showed in Table 3. Gestational age affected significantly copper (β = 0.652, p＜0.001) and zinc levels (β = −0.712, p＜0.001), but the effects were the opposite. Specifically, a positive relationship was observed between copper and gestational age, while a negative relationship was between zinc and gestational age. In the subgroup analysis, the effects of gestational age on copper and zinc varied across trimesters. For copper, except for 2nd trimester, copper levels increased with advancing gestational age, with the strongest effects on copper levels in the 1st trimester (β = 0.510, p＜0.001). For zinc, the strongest effect of gestational age on zinc levels was in the 2nd trimester (β = −0.422, p＜0.001). Additionally, maternal age and parity had a weak impact on the copper and zinc. Maternal age slightly affected copper levels in the 1st trimester (β = 0.140, p = 0.009). In addition, parity had little affect on copper (β = 0.142, p = 0.038) and zinc (β = −0.012, p = 0.048) in the 2nd trimester.

**Table 3:** Multivariate analysis of impact of copper and zinc during pregnancy.

			Copper		Zinc	
			**Coefficients(**β**)**	**P value**	**Coefficients(**β)	**P value**
**Total**	**Gestational Age**		0.652	＜0.001	−0.712	＜0.001
	**Maternal Age**		0.002	0.960	−0.020	0.466
	**Parity**	1	0.011	0.707	0.003	0.927
		2	0.030	0.309	−0.039	0.150
		0	Reference	Reference	Reference	Reference
**1**st **trimester**	**Gestational Age**		0.510	＜0.001	−0.208	＜0.001
	**Maternal Age**		0.140	0.009	−0.090	0.140
	**Parity**	1	−0.096	0.073	0.027	0.654
		2	0.013	0.805	−0.032	0.590
		0	Reference	Reference	Reference	Reference
**2**nd **trimester**	**Gestational Age**		0.081	0.204	−0.422	＜0.001
	**Maternal Age**		−0.113	0.102	0.041	0.517
	**Parity**	1	0.142	0.038	−0.024	0.695
		2	0.047	0.470	−0.012	0.048
		0	Reference	Reference	Reference	Reference
**3**rd **trimester**	**Gestational Age**		0.211	0.005	0.040	0.592
	**Maternal Age**		−0.088	0.261	0.036	0.651
	**Parity**	1	0.098	0.209	−0.046	0.563
		2	0.036	0.629	0.062	0.411
		0	Reference	Reference	Reference	Reference

Coefficients were presented as standardized regression coefficient.

## 4 Establishment RIs for copper and zinc levels during pregnancy

### 4.1 Establishment of trimester-specific RIs for copper and zinc by non-parametric method

Given that the differences of copper and zinc levels in three trimesters, we utilized the non-parametric method to establish trimester-specific RIs. The results are showed in Table 4. The RIs for copper were 750.15–1928.65 μg/L, 1311.37–2419.46 μg/L, and 1346.15–2411.85 μg/L in the 1st, 2nd, 3rd trimester, while RIs for zinc were 611.77–1100.45 μg/L, 471.01–861.10 μg/L, and 436.88–767.97 μg/L, respectively.

**Table 4:** RIs for copper and zinc in different trimesters by non-parametric method.

	N	Copper (μg/L)		Zinc (μg/L)	
		LL (90%CI)	UL (90%CI)	LL (90%CI)	UL (90%CI)
1st trimester	280	750.15 (638.00,810.00)	1928.65 (1798.00,2186.00)	611.77 (524.00,654.00)	1100.45 (1032.00,1191.00)
2nd trimester	248	1311.37 (1047.00,1360.00)	2419.46 (2264.00,2531.00)	471.01 (452.00,498.00)	861.10 (834.00,961.00)
3rd trimester	182	1346.15 (1154.00,1383.00)	2411.85 (2319.00,2630.00)	436.88 (398.00,466.00)	767.97 (741.00,856.00)

Abbreviation: LL: low limit of the reference interval; UL: upper limit of the reference interval.

### 4.2 Establishment of continuous RIs for copper and zinc by GAMLSS model

We utilized GAMLSS model to visualize trend in copper and zinc during pregnancy and established continuous RIs for them. Data from pregnant women with gestational aged 1–40 weeks were included in the curve estimation. The gestational age based continuous RIs were presented in Table 5 and the centile values (25th, 50th,75th) are shown in Supplement Table 1 and Supplement Fig. 1 in detail. Copper levels increased sharply until early 2nd trimester (around 18week) and then remained stable in the 2nd trimester. Copper levels decreased slightly in the early 3rd trimester (about 28–32 week) while increased in the late 3rd trimester (after 32 week), peaking at delivery. In contrast to copper, zinc levels showed a downward trend until 3rd trimester and then remained steady until delivery.

**Table 5:** Next-generation RIs for copper and zinc during pregnancy by GAMLSS model.

	Gestational Age	Total number	Copper (μg/L)		Zinc (μg/L)	
			P2.5	P97.5	P2.5	P97.5
1st trimester	1-	34	531.92	1082.20	676.10	1167.13
	2-		575.35	1170.54	668.89	1154.68
	3-		618.84	1259.03	661.689	1142.24
	4-		662.75	1348.36	654.489	1129.81
	5-		707.60	1439.61	647.299	1117.40
	6-	204	754.40	1534.83	640.03	1104.86
	7-		803.87	1635.48	632.39	1091.68
	8-		855.14	1739.78	623.93	1077.08
	9-		906.65	1844.58	614.27	1060.40
	10-		956.94	1946.90	603.37	1041.58
	11-	69	1005.21	2045.10	591.45	1021.01
	12-		1051.04	2138.34	578.88	999.30
	13-		1093.46	2224.65	565.98	977.04
2nd trimester	14-		1131.40	2301.84	553.12	954.84
	15-		1164.10	2368.35	540.60	933.22
	16-	83	1191.26	2423.61	528.80	912.85
	17-		1212.94	2467.73	518.00	894.21
	18-		1229.16	2500.73	508.27	877.42
	19-		1240.10	2522.98	499.49	862.26
	20-		1246.37	2535.74	491.43	848.34
	21-	97	1249.05	2541.19	483.84	835.24
	22-		1249.18	2541.46	476.68	822.88
	23-		1247.55	2538.15	469.92	811.21
	24-		1244.66	2532.25	463.60	800.30
	25-		1240.81	2524.43	457.88	790.42
	26-	108	1236.20	2515.04	452.82	781.69
	27-		1231.49	2505.47	448.68	774.54
3rd trimester	28-		1227.51	2497.37	445.63	769.27
	29-		1225.06	2492.37	443.61	765.78
	30-		1224.85	2491.95	442.49	763.86
	31-	94	1227.35	2497.04	442.12	763.22
	32-		1232.66	2507.85	442.28	763.49
	33-		1240.35	2523.50	442.82	764.42
	34-		1250.03	2543.19	443.61	765.78
	35-		1261.37	2566.26	444.55	767.42
	36-	21	1274.05	2592.05	445.63	769.27
	37-		1287.64	2619.70	446.78	771.27
	38-		1301.68	2648.26	448.02	773.40
	39-		1315.82	2677.04	449.28	775.58
	40-		1329.98	2705.85	450.55	777.77

**Fig. 3:**
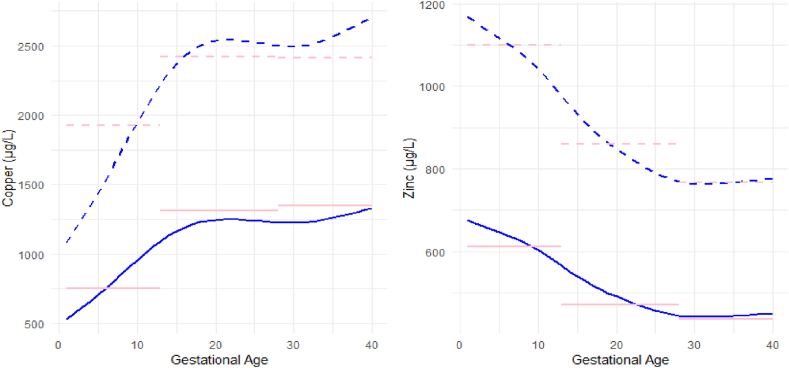
Comparison between discrete RIs and next-generation RIs for copper and zinc. Figures show RIs for copper (left) and zinc (right). Blue lines present next-generation RI and pink lines represent discrete RIs for copper and zinc. Solid lines: 2.5 percentile (P2.5); dashed lines: 97.5 percentiles (P97.5).

The continuous RIs were graphed with discrete RIs to direct visual comparison and shown in Fig. 3. It can be observed that there is a great discrepancy on RIs established by these two methods.

## 5 Discussion

Copper and zinc are two crucial trace elements that interact with each other, playing an important role in maintaining a normal pregnancy and the development of the fetus. During pregnancy, due to changes in physiological status, dietary changes, as well as the requirements from the mother herself and the fetus, copper and zinc levels change dramatically. Herein, establishing RIs for copper and zinc in pregnant women is essential for monitoring the status of copper and zinc, as well as timely diagnosing copper and zinc abnormalities. In this study, we compared levels of copper and zinc between pregnant women across trimesters and non-pregnant women. Moreover, we explored the effects of multiple factors (gestational age, maternal age, and parity) on copper and zinc levels. More importantly, trimester-specific discrete RIs by non-parametric method and Hoffmann method, as well as next-generation RIs based on gestational age by GAMLSS model were established.

Comparing copper and zinc levels between pregnant women and non-pregnant women, copper levels were higher in pregnant women, while zinc levels were lower in pregnant women, consistent with previous studies [5,24,25]. Moreover, levels of copper and zinc differed across trimesters.

Furthermore, we investigated several factors (gestational age, maternal age, and parity) that may influence levels of copper and zinc during pregnancy by multivariate regression. As expected, gestational age was significantly associated with copper and zinc levels, though effects varied among trimesters, suggesting the dynamic trend of copper and zinc should be elucidated more profoundly. In addition to the influence of gestational age on copper and zinc, other factors, maternal age and parity, also had an effect on copper or zinc levels in certain trimesters, but to a lesser degree.

Due to the difficulty of recruiting healthy pregnant women, only several articles established RIs for copper and zinc during pregnancy [5,6]. Furthermore, with changes in the methodology of detection of plasma copper and zinc, there is a need to establish RIs based on ICP-MS. In this study, we utilized the non-parametric method to establish trimester-specific RIs for copper and zinc by retrospectively collecting data from healthy pregnant women. In comparison trimester-specific in this study with RIs in previous studies [5,6], showing wide variation in RIs across studies, which may be attributed to differences in geography, detection method, definition of trimesters, and sample type. Therefore, it is crucial to establish specific RIs based on different laboratories.

More importantly, given the considerable effects of gestational age on copper and zinc, we further plotted the trend in copper and zinc levels in detail and established next-generation RIs by the GAMLSS model. Based on the model, it can be observed that copper levels underwent a dramatic fluctuation, with two significant increases during pregnancy, which may be due to the increment in estrogen [26], as well as stress response in the delivery. The trend in copper in this research was consistent with a previous study [27] but differed from most previous studies, which showed a gradual increase during pregnancy [[28], [29], [30], [31]] and other articles showed a plateau from the 2nd trimester and 3rd trimester [5] or even a slight increase in the 3rd trimester [25,32]. However, the trend of copper in previous studies was concluded by trimester partition, which may overlook small changes in copper levels. Conversely, zinc levels exhibited a downward trend during pregnancy. A decrease in zinc levels may be associated with a decrease in albumin concentration caused by the expansion of blood volume and the antagonist effect between copper and zinc [3,25]. The downward trend of zinc levels was consistent with previous studies [[27], [28], [29], [30], [31]] [[27], [28], [29], [30], [31]].

There are some advantages in this study. Firstly, we investigated multiple factors that affect copper and zinc levels and their impact on copper and zinc levels in different trimesters, which may be useful for elucidating mechanisms of changes in copper and zinc levels during pregnancy. Secondly, it is the first time to utilize the GAMLSS model to visualize trends in copper and zinc during pregnancy and establish gestational age next-generation RIs. The next-generation model depicts changes in copper and zinc in detail and can help doctors to monitor copper and zinc status accurately. Last but not least, data used for establishing RIs in this study was through retrospective collection of real-world data, which is time-saving and economic. However, the limitations of this study also can not be ignored. Firstly, according to the recommendation in the guideline [33], ultrasound data, plus LMP data, were the preferred method to estimate gestational age. However, there were some pregnant women (＜5 %)lacking ultrasound data, of whom the estimation of gestational age was based on the LMP data. Secondly, we are unable to collect some information such as intake of nutrients supplements, which may also have an effect on copper and zinc levels. However, we employed Hoffmann algorithm [20] to establish RIs(Supplement Table 2) in the whole pregnant women (N = 1210), a algorithm can distinguish distribution of healthy population from mixed distribution, to investigate the possible influence of nutrient on RIs in this study. RIs calculated by Hoffmann algorithm were similar to RIs by non-parametric method in this studies, proving that the effect of nutrient on enrolled pregnant women was negligible and accuracy of RIs in this study. Moreover, enrolling large-scale data from pregnant women in the future study is also necessary to verify the robustness, accuracy, and applicability of RIs established in this study.

## 6 Conclusion

Taken together, significantly higher copper levels and lower zinc levels were observed in pregnant women than in non-pregnant women. Additionally, levels of copper and zinc vary across trimesters. Gestational age has a remarkable influence on copper and zinc, while parity and maternal age only have a weak effect. Finally, next-generation RIs by GAMLSS model provide a detailed description of changes in copper and zinc and the discrepancy between discrete RIs and next-generation RIs during pregnancy suggests that next-generation RIs should be considered in the future to monitor copper and zinc status, as well as help in timely diagnosing copper and zinc disorders.

## Data availability

The authors declare that all data generated or analyzed during this study are included in this published article.

## CRediT authorship contribution statement

**Lihua Guan:** Writing – review & editing, Writing – original draft, Formal analysis. **Chaochao Ma:** Writing – review & editing, Software, Methodology. **Liling Lin:** Writing – review & editing, Supervision. **Ling Qiu:** Writing – review & editing, Supervision, Funding acquisition, Conceptualization.

## Declaration of competing interest

The authors declare that they have no known competing financial interests or personal relationships that could have appeared to influence the work reported in this paper.
