# Supplementary material for: Establishment of discrete reference interval and next-generation reference interval for copper and zinc during pregnancy using real-world data
Source: Heliyon. 2024 Jun 29;10(13):e33856. doi: 10.1016/j.heliyon.2024.e33856 (PMC11268205; doi:10.1016/j.heliyon.2024.e33856)
Supplement: Multimedia component 1 [file mmc1.doc]

| **Table S1 Centile values of copper and zinc by GAMLSS model** | | | | | | | | | | |
| --- | --- | --- | --- | --- | --- | --- | --- | --- | --- | --- |
| Gestational Age | Copper(µg/L) | | | | | Zinc(µg/L) | | | | |
|  | 2.5^th^ | 25^th^ | 50^th^ | 75^th^ | 97.5^th^ | 2.5^th^ | 25^th^ | 50^th^ | 75^th^ | 97.5^th^ |
| 1- | 531.92 | 686.19 | 766.44 | 854.42 | 1082.20 | 676.10 | 824.84 | 903.87 | 986.76 | 1167.13 |
| 2- | 575.35 | 742.21 | 829.01 | 924.17 | 1170.54 | 668.89 | 816.04 | 894.23 | 976.24 | 1154.68 |
| 3- | 618.84 | 798.31 | 891.68 | 994.03 | 1259.03 | 661.68 | 807.25 | 884.60 | 965.72 | 1142.24 |
| 4- | 662.75 | 854.95 | 954.94 | 1064.56 | 1348.36 | 654.48 | 798.47 | 874.97 | 955.21 | 1129.81 |
| 5- | 707.60 | 912.82 | 1019.57 | 1136.61 | 1439.61 | 647.29 | 789.69 | 865.36 | 944.72 | 1117.40 |
| 6- | 754.40 | 973.19 | 1087.01 | 1211.78 | 1534.83 | 640.03 | 780.83 | 855.64 | 934.11 | 1104.86 |
| 7- | 803.87 | 1037.01 | 1158.29 | 1291.25 | 1635.48 | 632.39 | 771.52 | 845.44 | 922.98 | 1091.68 |
| 8- | 855.14 | 1103.14 | 1232.15 | 1373.59 | 1739.78 | 623.93 | 761.20 | 834.13 | 910.63 | 1077.08 |
| 9- | 906.65 | 1169.60 | 1306.38 | 1456.34 | 1844.58 | 614.27 | 749.41 | 821.21 | 896.53 | 1060.40 |
| 10- | 956.94 | 1234.47 | 1378.84 | 1537.12 | 1946.90 | 603.37 | 736.11 | 806.64 | 880.62 | 1041.58 |
| 11- | 1005.21 | 1296.74 | 1448.39 | 1614.65 | 2045.10 | 591.45 | 721.57 | 790.71 | 863.23 | 1021.01 |
| 12- | 1051.04 | 1355.86 | 1514.42 | 1688.27 | 2138.34 | 578.88 | 706.23 | 773.90 | 844.87 | 999.30 |
| 13- | 1093.46 | 1410.59 | 1575.55 | 1756.41 | 2224.65 | 565.98 | 690.50 | 756.66 | 826.05 | 977.04 |
| 14- | 1131.40 | 1459.53 | 1630.22 | 1817.35 | 2301.84 | 553.12 | 674.81 | 739.46 | 807.28 | 954.84 |
| 15- | 1164.10 | 1501.70 | 1677.32 | 1869.87 | 2368.35 | 540.60 | 659.53 | 722.72 | 789.00 | 933.22 |
| 16- | 1191.26 | 1536.74 | 1716.46 | 1913.50 | 2423.61 | 528.80 | 645.13 | 706.95 | 771.78 | 912.85 |
| 17- | 1212.94 | 1564.72 | 1747.71 | 1948.33 | 2467.73 | 518.00 | 631.96 | 692.51 | 756.02 | 894.21 |
| 18- | 1229.16 | 1585.64 | 1771.08 | 1974.39 | 2500.73 | 508.27 | 620.10 | 679.51 | 741.82 | 877.42 |
| 19- | 1240.10 | 1599.75 | 1786.84 | 1991.95 | 2522.98 | 499.49 | 609.38 | 667.77 | 729.01 | 862.26 |
| 20- | 1246.37 | 1607.84 | 1795.87 | 2002.02 | 2535.74 | 491.43 | 599.54 | 656.99 | 717.24 | 848.34 |
| 21- | 1249.05 | 1611.30 | 1799.74 | 2006.33 | 2541.19 | 483.84 | 590.29 | 646.84 | 706.16 | 835.24 |
| 22- | 1249.18 | 1611.47 | 1799.93 | 2006.54 | 2541.46 | 476.68 | 581.55 | 637.27 | 695.71 | 822.88 |
| 23- | 1247.55 | 1609.37 | 1797.58 | 2003.92 | 2538.15 | 469.92 | 573.30 | 628.23 | 685.84 | 811.21 |
| 24- | 1244.66 | 1605.63 | 1793.40 | 1999.27 | 2532.25 | 463.60 | 565.59 | 619.78 | 676.62 | 800.30 |
| 25- | 1240.81 | 1600.67 | 1787.86 | 1993.09 | 2524.43 | 457.88 | 558.61 | 612.13 | 668.27 | 790.42 |
| 26- | 1236.20 | 1594.71 | 1781.21 | 1985.68 | 2515.04 | 452.82 | 552.44 | 605.37 | 660.89 | 781.69 |
| 27- | 1231.49 | 1588.65 | 1774.44 | 1978.13 | 2505.47 | 448.68 | 547.39 | 599.83 | 654.84 | 774.54 |
| 28- | 1227.51 | 1583.51 | 1768.70 | 1971.73 | 2497.37 | 445.63 | 543.66 | 595.75 | 650.39 | 769.27 |
| 29- | 1225.06 | 1580.34 | 1765.16 | 1967.79 | 2492.37 | 443.61 | 541.20 | 593.05 | 647.44 | 765.78 |
| 30- | 1224.85 | 1580.08 | 1764.86 | 1967.45 | 2491.95 | 442.49 | 539.84 | 591.56 | 645.81 | 763.86 |
| 31- | 1227.35 | 1583.30 | 1768.47 | 1971.47 | 2497.04 | 442.12 | 539.39 | 591.07 | 645.28 | 763.22 |
| 32- | 1232.66 | 1590.16 | 1776.12 | 1980.01 | 2507.85 | 442.28 | 539.58 | 591.28 | 645.50 | 763.49 |
| 33- | 1240.35 | 1600.08 | 1787.20 | 1992.36 | 2523.50 | 442.82 | 540.24 | 592.00 | 646.29 | 764.42 |
| 34- | 1250.03 | 1612.56 | 1801.15 | 2007.91 | 2543.19 | 443.61 | 541.20 | 593.05 | 647.44 | 765.78 |
| 35- | 1261.37 | 1627.19 | 1817.49 | 2026.12 | 2566.26 | 444.55 | 542.35 | 594.32 | 648.82 | 767.42 |
| 36- | 1274.05 | 1643.55 | 1835.75 | 2046.48 | 2592.05 | 445.63 | 543.66 | 595.75 | 650.39 | 769.27 |
| 37- | 1287.64 | 1661.08 | 1855.34 | 2068.31 | 2619.70 | 446.78 | 545.07 | 597.30 | 652.08 | 771.27 |
| 38- | 1301.68 | 1679.19 | 1875.56 | 2090.86 | 2648.26 | 448.02 | 546.58 | 598.95 | 653.88 | 773.40 |
| 39- | 1315.82 | 1697.44 | 1895.95 | 2113.59 | 2677.04 | 449.28 | 548.12 | 600.64 | 655.72 | 775.58 |
| 40- | 1329.98 | 1715.70 | 1916.35 | 2136.33 | 2705.85 | 450.55 | 549.67 | 602.33 | 657.57 | 777.77 |


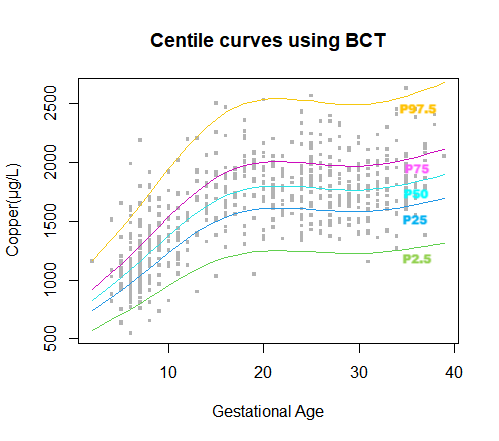

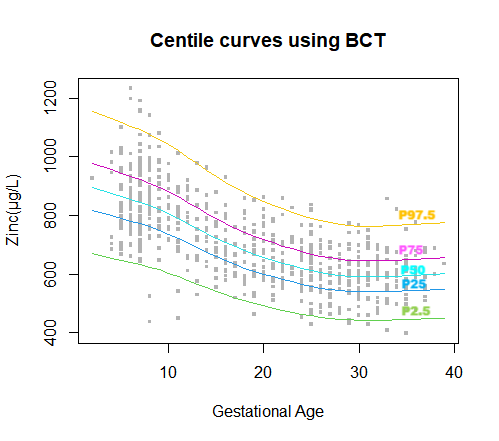


**Figure S1 Trend in copper and zinc by GAMLSS model**

| **Table S2 RIs for copper and zinc in different trimesters by Hoffmann algorithm** | | | | |
| --- | --- | --- | --- | --- |
|  | Copper(μg/L) | | Zinc(μg/L) | |
|  | LL | UL | LL | UL |
| 1^st^ trimester | 737.93 | 1998.13 | 634.20 | 1064.62 |
| 2^nd^ trimester | 1296.76 | 2351.90 | 486.44 | 879.46 |
| 3^rd^ trimester | 1520.09 | 1908.52 | 462.20 | 796.98 |
